# Vertically Aligned Carbon Nanotubes Grown on Copper Foil as Electrodes for Electrochemical Double Layer Capacitors

**DOI:** 10.3390/nano15191506

**Published:** 2025-10-01

**Authors:** Chinaza E. Nwanno, Ram Chandra Gotame, John Watt, Winson Kuo, Wenzhi Li

**Affiliations:** 1 Department of Physics, Florida International University, Miami, FL 33199, USA; 2 Centre for Integrated Nanotechnologies, Los Alamos National Laboratory, Los Alamos, NM 87545, USA

**Keywords:** VACNTs, PECVD, copper substrate, EDLC, specific capacitance, supercapacitor

## Abstract

This study reports a binder-free, catalyst-free method for fabricating vertically aligned carbon nanotubes (VACNTs) directly on copper (Cu) foil using plasma-enhanced chemical vapor deposition (PECVD) for electrochemical double-layer capacitor (EDLC) applications. This approach eliminates the need for catalyst layers, polymeric binders, or substrate pre-treatments, simplifying electrode design and enhancing electrical integration. The resulting VACNTs form a dense, uniform, and porous array with strong adhesion to the Cu substrate, minimizing contact resistance and improving conductivity. Electrochemical analysis shows gravimetric specific capacitance (C_grav_) and areal specific capacitance (C_areal_) of 8 F g^−1^ and 3.5 mF cm^−2^ at a scan rate of 5 mV/s, with low equivalent series resistance (3.70 Ω) and charge transfer resistance (0.48 Ω), enabling efficient electron transport and rapid ion diffusion. The electrode demonstrates excellent rate capability and retains 92% of its initial specific capacitance after 3000 charge–discharge cycles, indicating strong cycling stability. These results demonstrate the potential of directly grown VACNT-based electrodes for high-performance EDLCs, particularly in applications requiring rapid charge–discharge cycles and sustained energy delivery.

## 1. Introduction

The growing demand for efficient and reliable energy storage systems has intensified research into advanced materials that meet the high-performance requirements of modern electronic devices, renewable energy systems, and electric vehicles (EVs) [1,2]. Among various energy storage technologies, EDLCs have garnered significant attention due to their ability to deliver rapid charge and discharge cycles, long cycle life, and high-power density. The EDLCs store energy through the electrostatic accumulation of charges at the interface between the electrode and the electrolyte, forming an electrical double layer [3,4]. This mechanism, which involves no faradaic (chemical) reactions, allows for fast charge transfer and high reversibility, making EDLCs particularly suitable for applications requiring high power density and longevity.

A critical component of EDLCs is the electrode material, which directly influences the overall performance, such as capacitance, energy density, and cycle stability. Carbon-based materials, particularly carbon nanotubes (CNTs), have emerged as promising candidates due to their unique structural, electrical, and mechanical properties [5,6]. VACNTs, consisting of well-defined arrays of individual CNTs aligned perpendicular to the substrate surface, are considered more suitable for fabricating high-performance EDLCs. Compared to their randomly oriented counterparts, the VACNTs provide better access to their entire surface area, while the well-defined spaces between individual nanotubes act as ionic pathways, enhancing ion diffusion rates [7,8]. For example, Zhang et al. [9] compared the electrochemical properties of aligned CNTs (ACNTA) and entangled CNTs (ECNT) in their study, finding that the ACNTA exhibited higher specific capacitance, better rate capability, and lower equivalent series resistance (ESR) than the ECNT. They attributed the superior performance of the ACNTA electrode to its larger pore size and more regular structure, which contrasts with the more irregular arrangement of the ECNT electrode.

Traditionally, electrode fabrication for EDLCs involved growing CNTs on semiconducting or non-conductive substrates, which then required additional steps to transfer to prepare CNTs for use on the conductive current collector [10]. This process often included preparing a slurry by mixing the CNTs with conductive additives and binders. The resulting paste was then applied to a conductive substrate, such as aluminum (Al) or copper (Cu), as the current collector. However, this method introduced several issues, including increased contact resistance between the CNTs and the current collector, which could reduce overall conductivity and impede efficient ion and electron transport [11]. The binders also added weight and introduced an additional barrier, limiting the electrode’s ability to support the rapid charge and discharge cycles characteristic of EDLC.

To overcome these limitations, recent research has shifted toward directly growing VACNTs on conductive metal substrates like Cu [12], Al [13], stainless steel (SS) [14], and Inconel [15]. Direct growth of VACNTs on metal substrates eliminates the need for binders and slurry preparation, reducing the complexity of electrode fabrication and enhancing performance. Although significant progress has been made, most of these efforts still require depositing catalyst layers such as Iron (Fe), Cobalt (Co), and Nickel (Ni) on the conductive metal substrates. A major drawback of this approach is the diffusion of the catalyst film into the substrate, which hinders the growth of the CNTs. This issue occurs because the high surface energy of metal substrates prevents the catalyst film from properly dewetting and forming nanoparticles that are required for CNT growth [16]. To address this issue, an intermediate layer is often added between the catalyst film and the metal substrate; however, this additional layer increases the weight of the electrode and interfacial resistance, which can adversely affect performance in EDLC applications.

Studies have shown that this issue can be solved by choosing metal substrates with catalytic properties for CNT growth, like SS, Ni foam, Al, and Cu, which can also act as current collectors [17,18,19]. Avasthi et al. [20] reported the growth of aligned CNTs on SS for flexible supercapacitor electrodes with a high specific capacitance of 5.99 mF ${cm}^{-2}$ at 1.67 mA ${cm}^{-2}$ and capacity retention of 140% after 2000 cycles. The successful growth of the aligned CNTs on the stainless steel is believed to be because of the catalytic property of the constituent Fe in the SS, which is a well-known catalyst for CNT growth. Similarly, Lei et al. [17] reported a high specific capacitance of 11 mF ${cm}^{-2}$ and a high capacity retention of 97% after 5000 cycles at a scan rate of 0.5 mA cm^−2^, a low series and charge transfer resistance of 0.39 Ω cm^−2^ and 2.97 Ω cm^−2^ for CNTs directly grown on stainless steel without additional layers. Isacfranklin et al. [18] reported an impressive specific capacitance of 250.51 F g^−1^, along with high energy and power densities of 68.19 Wh kg^−1^ and 2799.77 W kg^−1^, respectively, for a binder-free CNT electrode directly synthesized on a Ni foam substrate. Furthermore, the electrode demonstrated excellent capacity retention of 99.68% even after 10,000 cycles. They attributed the superb performance of the electrode to the intimate contact between the CNTs and the Ni foam current collector. Querne et al. [21] also conducted a comparative study of high-density VACNTs grown directly (without extra layers) on different grades of aluminum for EDLC applications. The VACNTs delivered a high specific capacitance of 45 F g^−1^ and a high capacity retention of 90% at a current density of 13 A g^−1^.

On the other hand, copper has long been considered an ideal current collector for energy applications due to its excellent electrical and thermal conductivity and low cost [22]. Growing VACNTs directly on Cu for electrode fabrication would minimize contact resistance, ensure maximum conductivity, and improve charge transport properties. These are crucial for the rapid charge and discharge cycles typical of EDLCs. This strategy also provides the electrode with mechanical stability, which is vital for maintaining consistent performance during repeated charge–discharge cycles. Furthermore, using Cu as a substrate enables easier integration of VACNT-based electrodes into existing manufacturing processes for commercial supercapacitors, accelerating the production of high-performance energy storage devices.

However, despite these advantages and the massive potential of Cu/VACNT-based electrodes, there is a shortage of studies on the electrochemical performance of VACNTs grown directly on copper without intermediate layers. This gap is primarily due to copper’s poor catalytic behavior during CNT growth compared to other metals like nickel and iron [23]. Copper-filled 3d orbitals prevent the formation of strong covalent bonds with hydrocarbon molecules, making direct CNT growth on copper substrates challenging. Thus, existing studies rely on the prior deposition of additional layers onto the Cu substrate to achieve CNT growth. For instance, Atthipalli et al. [24] grew multi-walled CNTs (MWCNTs) on bulk copper wafers by first depositing Ni film using CVD. Sepahvand et al. [25] obtained dense arrays of VACNTs by depositing Ni and chromium (Cr) layers as a catalyst and diffusion barrier layers, respectively. Lahiri et al. achieved CNT growth on copper by depositing Titanium (Ti) as an intermediate layer and Ni as a catalyst [26]. Céspedes et al. [27] reported densely packed mats of CNTs by coating Titanium nitride (TiN) on copper to prevent the diffusion of the Fe catalyst during growth. They also reported no CNT growth on pure copper substrate. Li et al. [28] employed an e-beam lithography technique to deposit layers of Titanium (Ti) and nickel-chromium (Ni-Cr) between copper substrates and the alumina buffer layer before depositing the Fe catalyst. This was performed to reduce surface cracking and defects from the copper substrates and facilitate the growth of VACNTs. All these studies mentioned above relied on the prior deposition of multiple layers on the copper substrate to achieve successful growth of CNTs. Aside from the increased interfacial resistance, the method is tortuous and costly.

In this work, we demonstrate a simple, binder-free method to fabricate EDLC electrodes by directly growing VACNTs on thin Cu foil substrates using the PECVD technique reported in our previous works [29,30]. PECVD was chosen for its ability to enable CNT growth and alignment even on poorly catalytic substrates like Cu, due to ion-assisted growth and enhanced nucleation under plasma conditions. The method eliminates the need of binders, additional metallic catalysts, or substrate etching steps, simplifying fabrication process and minimizing contact resistance. The resulting electrode demonstrated excellent rate capability and cycling stability, along with low equivalent series and charge transfer resistances, owing to the well-ordered porous structure of the VACNT array and the direct integration of the nanotubes with the copper current collector. To our knowledge, this is one of the few reports demonstrating VACNT growth on Cu foil by PECVD for EDLC applications.

## 2. Experimental

### 2.1. Synthesis of VACNTs

**Figure 1:**
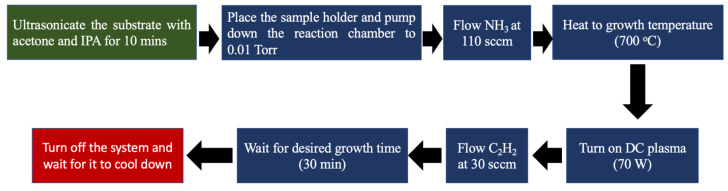
Schematic representation of the synthesis procedure for VACNTs on Cu foil using PECVD.

The step-by-step synthesis process is illustrated in Figure 1. VACNTs were grown directly on Cu foils of 0.2 mm thickness using a PECVD technique. Before the synthesis process, a piece of Cu foil was cut into square shapes of 1.5 cm × 1.5 cm to be used as substrates for the VACNT growth. The Cu substrates were ultrasonicated in acetone and isopropyl alcohol (IPA) for 10 min each to remove surface contaminants. The substrates were allowed to dry in the open air before being loaded into the PECVD chamber for VACNT growth. The chamber was evacuated to a base pressure of 0.01 Torr. During the temperature ramp to 700 °C at 50 °C/min, ammonia (NH_3_) was introduced at 110 sccm and maintained at 7 Torr. The NH_3_ acted as an etching agent, creating uniform catalytic sites necessary for nanotube nucleation. Once the growth temperature was reached, DC plasma was ignited at 70 W, and acetylene (C_2_H_2_) was introduced at 30 sccm as the carbon source. Growth continued for 30 min before the system was shut down and allowed to cool to room temperature.

### 2.2. Material Characterization

#### 2.2.1. Structural Characterization

The surface morphology of the VACNTs was characterized by a field emission scanning electron microscope (FESEM; JEOL, Tokyo, Japan) operated at an accelerating voltage of 15 kV. A transmission electron microscope (TEM; ThermoFisher Titan 80-300, Waltham, MA, USA) operated at 300 kV was used to examine the submicron structures of the as-synthesized VACNTs. The sample was prepared for the TEM characterization by gently scraping the VACNTs off the Cu foil substrate using a surgical blade. The scraped VACNTs were then flushed off the blade with a few drops of alcohol. Raman spectroscopy (UniRAM-3500) with 632.8 nm laser excitation was used to investigate the quality and purity of the VACNTs. The X-ray Diffraction (XRD) pattern was obtained using the Siemens Diffractometer D5000 (Munich, Germany) with Cu Kα radiation (λ=1.54 Å) operated in a θ−2θ configuration. Data was collected over the 2θ range of 20–70° with a step size of 0.02° and a scan speed of 0.5° min^−1^. To prepare the sample for XRD analysis, the VACNT film was mechanically detached from the Cu foil using double-sided Kapton tape. The freestanding CNT layer adhered to the tape was then mounted for analysis. This procedure ensured that the XRD spectrum represents only the VACNTs. The functional groups present on the VACNT surface were characterized using an Agilent Cary 660 FTIR spectrometer equipped with an attenuated total reflectance (ATR) accessory. Solid VACNT samples were gently scraped from the Cu foil using a clean plastic tweezer and transferred directly onto the diamond ATR crystal. The anvil pressure arm was then lowered to ensure firm contact between the CNT layer and the crystal. Spectra were recorded in the 4000–500 ${cm}^{-1}$ range at a resolution of 4 ${cm}^{-1}$, averaging 32 scans per measurement.

#### 2.2.2. Electrochemical Characterization

All electrochemical measurements were carried out in an electrochemical analyzer (CHI660E workstation; CH Instruments Inc., Bee Cave, TX, USA) using a three-electrode standard cell in 1M aqueous phosphate-buffer solution (PBS) as electrolyte at room temperature. The potentials and currents were measured with respect to Ag/AgCl (1M Na_2_SO_4_) as the reference electrode. The as-synthesized VACNTs on Cu foil and a Platinum (Pt) wire were used as the working and counter electrodes, respectively. CV measurements were performed at different scan rates, with the potential range from 0 to 0.9 V, to qualitatively evaluate the VACNT-based electrode’s charge–discharge behavior. Galvanostatic charge/discharge (GCD) measurements were performed at different current densities between the potential window of 0 and 0.9 V. The cyclability test was carried out by CV measurements at a low scan rate of 20 mV/s for 3000 cycles. Electrochemical impedance spectroscopy (EIS) was conducted in the frequency range of 0.1 Hz–300 kHz at a potential amplitude of 5 mV. To ensure the electrochemical analyzer and electrodes were fully activated, at least 20 CV cycles at a scan rate of 50 mV/s were performed before the electrochemical measurements. Both gravimetric (F $g^{-1}$) and areal (mF ${cm}^{-2}$) capacitances were calculated, with areal values obtained from the electrode area of 1.13 cm^−2^ and gravimetric values based on an active material mass of 0.5 mg.

## 3. Results and Discussion

### 3.1. VACNT Morphology

**Figure 2:**
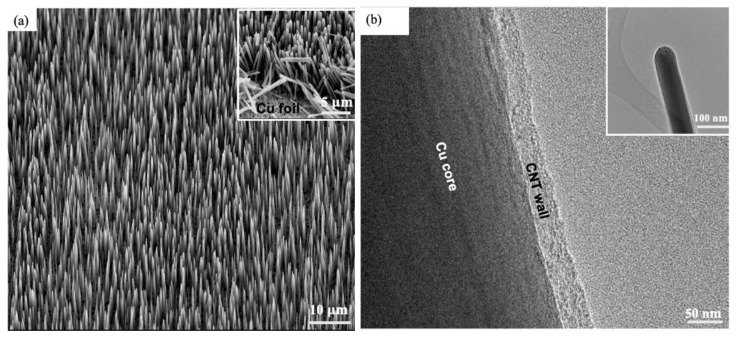
(**a**) SEM image showing the highly uniform VACNTs directly grown on Cu foil substrate. The inset provides a close-up view of a scraped portion of the VACNTs, showing their pore structure and direct attachment to the Cu foil. (**b**) HRTEM image displaying the multi-walled structure of an individual VACNT, with the inset showing an LRTEM image that reveals the uniform cylindrical shape of the VACNT.

The SEM image in Figure 2a shows the morphology of the VACNTs grown directly on the Cu substrate. The VACNTs form a highly uniform and dense array, with each nanotube exhibiting a well-aligned, vertical orientation relative to the substrate surface. The nanotubes have an average diameter of approximately 940 nm and a height of around 14 μm, forming a vertically oriented porous structure that promotes effective electrolyte diffusion and ion transport during electrochemical cycling. The inset of Figure 2a provides a close-up view of a portion of the scraped VACNTs, revealing the direct attachment of the roots of the VACNTs to the Cu substrate and the presence of a well-defined pore structure that is formed between the individual nanotubes. The presence of these pores is essential for rapid charge and discharge cycles in EDLCs and enhances the active surface area available for charge accumulation [31,32,33]. The well-preserved structure after mechanical removal, as seen in the inset of Figure 2a, suggests that the VACNTs maintain their structural integrity even under physical stress, further confirming their suitability as durable electrode materials.

Figure 2b, the high-resolution transmission electron microscopy (HRTEM) image, offers a closer look at an individual VACNT, revealing its multi-walled structure with well-defined concentric graphene layers. This multi-walled configuration contributes to the mechanical strength and durability of the VACNTs by enhancing their ability to sustain the structural integrity of the electrode during repeated charge–discharge cycles. The inset of Figure 2b offers a low-resolution transmission electron microscopy (LRTEM) view of the individual VACNT, providing valuable insight into its overall structure and dimensions. This LRTEM image clearly shows the cylindrical nature of the VACNT, with a consistent and uniform diameter along its length.

**Figure 3:**
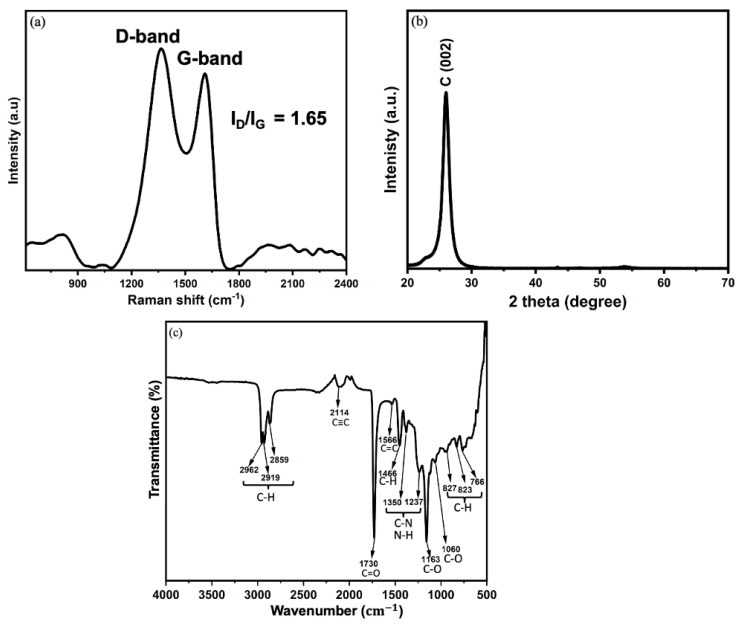
(**a**) Raman, (**b**) XRD, and (**c**) FTIR spectra of the as-synthesized VACNTs.

The Raman spectrum of the VACNTs shown in Figure 3a reveals two prominent peaks: the D-band at approximately 1339 cm^−1^ and the G-band at 1590 cm^−1^. The D-band corresponds to the breathing modes of sp^2^ carbon atoms in disordered or defect-rich regions of the CNT structure. Its presence indicates the existence of defects, such as vacancies, edge defects, or amorphous carbon within the VACNT array [34]. The G-band is associated with the stretching of sp^2^ carbon-carbon bonds in graphitic structures. It is indicative of the degree of graphitization and crystallinity of the VACNTs [35]. The intensity ratio of the D-band to the G-band (I_D_/I_G_) is a quantitative measure of the defect density and structural disorder in the VACNTs. An I_D_/I_G_ value of 1.65 suggests a high level of defects in the VACNT array, which is likely attributed to the high plasma intensity used during the CNT growth process [36]. Figure 3b presents the XRD pattern of the as-synthesized VACNTs. A prominent peak at 2θ = 26°, corresponding to the (002) plane of graphitic carbon confirms the formation of graphitized CNT walls [37]. FTIR analysis was performed to investigate the surface chemistry and bonding properties of the as-synthesized VACNTs. As shown in Figure 3c, the FTIR spectrum exhibits several absorption bands which indicate the presence of surface functionalities on the VACNTs. The peaks at 2962, 2919, and 2859 cm^−1^ correspond to asymmetric and symmetric stretching of aliphatic C–H bonds, suggesting the presence of residual hydrocarbons from the C_2_H_2_ decomposition or incomplete graphitization [38]. A weak peak at 2114 cm^−1^ is attributed to C≡C stretching, reflecting sp-hybridized carbon species or structural defects [39]. The strong peak at 1730 cm^−1^ is assigned to C=O stretching vibrations, likely arising from surface oxidation of the VACNTs during post-growth exposure to air [40]. A prominent peak at 1566 cm^−1^ corresponds to C=C stretching within the sp^2^-hybridized aromatic carbon lattice of the CNT walls [41]. The broad region between 1350 and 1237 cm^−1^ shows overlapping C–N and N–H bending vibrations, indicating nitrogen incorporation from NH_3_ during PECVD growth [42,43]. Peaks at 1163 and 1060 cm^−1^ correspond to C–O stretching, further supporting the surface oxidation that occurred after the growth process [44]. At lower wavenumbers, the peaks at 827, 823, and 766 cm^−1^ are associated with C–H out-of-plane bending, consistent with the behavior of aromatic and graphitic carbon structures [45].

### 3.2. Electrochemical Properties of VACNT Electrodes

**Figure 4:**
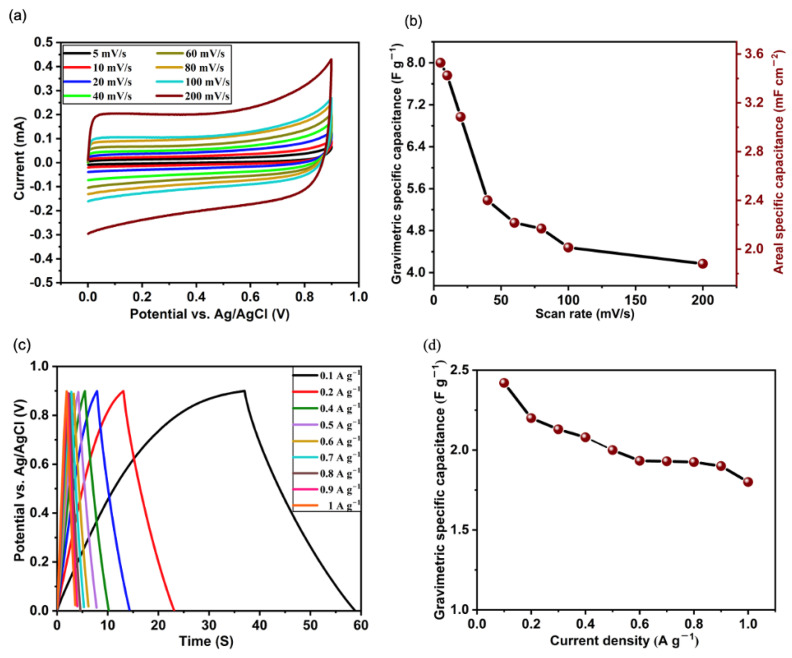
(**a**) Cyclic voltammetry curves of VACNT-based electrodes at various scan rates, (**b**) specific capacitance as a function of scan rate, (**c**) galvanostatic charge–discharge curves at different current densities, and (**d**) specific capacitance as a function of current density.

The CV plots shown in Figure 4a present the current response of the VACNT-based electrode at various scan rates, ranging from 5 mV/s to 200 mV/s. The CV curves exhibit a nearly rectangular shape across all scan rates, suggestive of the differential characteristic of a typical EDLC, where the capacitance itself depends on the applied terminal voltage [46,47]. The relatively stable and symmetrical nature of these curves across the different scan rates suggests that the VACNTs possess excellent charge storage capabilities with minimal resistive losses and fast charge–discharge kinetics across the potential window, which is essential for high-power applications. As the scan rate increases, there is a noticeable broadening of the CV curves, particularly at the higher scan rates (100 mV/s and 200 mV/s). This broadening can be attributed to the reduced time available for electrolyte ions to fully penetrate and interact with the entire surface area of the VACNTs [48,49]. Despite this, the VACNTs maintain a reasonably high level of capacitive behavior even at these elevated scan rates, indicating that the structure of the VACNTs facilitates rapid ion transport. The gravimetric (C_grav_) and areal specific capacitances (C_areal_) at different scan rates were calculated from the CV curves using Equation (1a,b) below [50]:

(1a)Cgrav=1m.ν.ΔV∫IVdV(1b)Careal=1A.ν.ΔV∫IVdV

where m = 0.5 mg is the mass loading of the VACNTs on the Cu foil, A = 1.13 ${cm}^{-2}$ is the area of the Cu current collector, ν is the potential scan rate, ΔV is the sweep potential window and $\int I_{\left(V\right)}$dV represents the integral of the area under the CV curves. The plot of specific capacitance versus scan rate shown in Figure 4b illustrates the dependence of the gravimetric and areal specific capacitances on scan rate. The gravimetric specific capacitance, which reflects the charge storage capability of the electrode material normalized by mass, is observed to be highest at the lowest scan rate (approximately 8 F $g^{-1}$ at 5 mV/s) while the area capacitance calculated by normalizing the capacitance by the area of the electrode (A = 1.13 cm^2^) is about 3.5 mF ${cm}^{-2}$. The higher capacitances at low scan rates can be attributed to the electrolyte ions having sufficient time to fully access and utilize the extensive surface area and pores of the VACNTs. However, as the scan rate increases, both the gravimetric and areal capacitances decrease, dropping to about 4 F $g^{-1}$ (1.8 mF ${cm}^{-2}$) at the highest scan rate of 200 mV/s. This reduction is a common phenomenon in porous electrode materials and is indicative of kinetic limitations in ion diffusion [51,52,53]. At higher scan rates, the time for ion diffusion is significantly reduced, leading to incomplete charge storage within the deeper or less accessible pores of the VACNTs. Consequently, only the outer regions of the VACNT structure are effectively utilized for charge storage at these higher rates, resulting in lower observed specific capacitance. However, as seen in Figure 4b, the VACNT electrode exhibits a gradual decline in capacitance at higher scan rates, which suggests that the VACNTs possess a hierarchical pore structure that still allows for some degree of ion penetration and charge storage even under fast cycling conditions.

The galvanostatic charge–discharge (GCD) curves shown in Figure 4c provide insights into the charge–discharge behavior of the VACNT-based electrode at various current densities, ranging from 0.1 A $g^{-1}$ to 1 A $g^{-1}$. Although the curve exhibits a noticeable asymmetry at the lowest current density of 0.1 A $g^{-1}$, which could be a result of the slow movement of ions within the electrolyte at this current density, the overall nearly isosceles triangular shape of the GCD curves across different current densities is indicative of the electrode’s capability to maintain its reversibility and good capacitive behavior under a range of operating conditions. The gravimetric specific capacitance at various current densities was calculated from Equation (2) below [54,55]:

(2) $$C_{\mathrm{grav}}=\frac{I\Delta t}{m\Delta V}$$

where C_grav_ is the gravimetric specific capacitance, I(A) is the discharge current, and m(g) is the mass of the VACNTs, Δt is the discharge time, and ΔV the potential window. As shown in Figure 4d, the gravimetric specific capacitance is highest at the lowest current density of 0.1 A $g^{-1}$, reaching approximately 2.4 F $g^{-1}$. As the current density increases, the gravimetric specific capacitance gradually decreases to about 1.8 F $g^{-1}$ at 1 A $g^{-1}$. Like the case with high scan rates, this reduction in specific capacitance with increasing current density is a typical behavior observed in porous electrodes and can be explained by the limited time available for ion transport and adsorption at higher current densities. At lower current densities, ions have more time to diffuse into the porous structure of the VACNTs, leading to higher specific capacitance. However, at higher current densities, the rapid charge–discharge cycles hinder the full utilization of the electrode material, resulting in lower capacitance values. The ability to retain a significant portion of its capacitance at higher current densities, as seen in Figure 4d, implies that the VACNT-based electrode exhibits good rate capability, which is a result of the well-developed pore structure and high available surface area of the VACNTs. This excellent behavior highlights the vast potential of the VACNT-based electrode in high-power applications requiring rapid charge and discharge cycles.

However, the low specific capacitance obtained from both the CV and GCD measurements can be attributed to the inherent hydrophobicity of the VACNTS used in the electrode, as shown by Ghai et al. [56]. CNTs, in general, exhibit a hydrophobic surface due to their nonpolar graphitic structure, which limits their interaction with aqueous electrolytes [57,58]. This wettability restricts the access of electrolyte ions to the CNT surface, reducing the effective surface area available for charge storage. Since the electrochemical double-layer capacitance is directly related to the accessible surface area of the electrode in contact with the electrolyte, poor wettability leads to incomplete penetration of electrolyte ions into the CNT network and, consequently, lower specific capacitance. To mitigate this issue, surface functionalization of CNTs is often employed. By introducing hydrophilic functional groups such as carboxyl (-COOH) or hydroxyl (-OH) groups on the surface of CNTs, their wettability is improved, enhancing the interaction with the electrolyte [59,60]. Another promising approach is the use of non-aqueous electrolytes (organic or ionic liquids) that enable wider potential windows and mitigate hydrophobicity effects [4]. Such systems have been shown to significantly enhance the capacitance of CNT-based electrodes [61,62,63].

**Figure 5:**
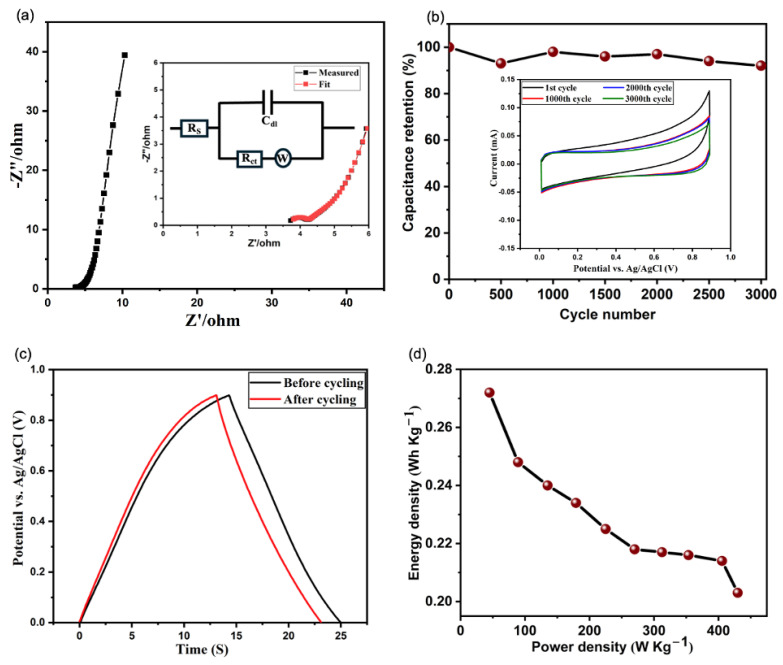
(**a**) Nyquist plot of the VACNT-based electrode (the inset is the equivalent circuit model from the fitted plot), (**b**) Capacitance retention of the VACNT-based electrode over 3000 cycles at a constant scan rate of 20 mV/s (the inset contains the CVs of the 1st, 1000th, 2000th, and 3000th cycles), (**c**) GCD curves at 0.2 A $g^{-1}$ before and after 3000 CV cycles, and (**d**) Ragone plot of the VACNT-based electrode.

Figure 5a shows the Nyquist plot of the VACNT-based electrode. The inset includes the measured and the fitted data, along with the equivalent circuit model of the Nyquist plot. The y-axis (Z″) and the x-axis (Z′) are the imaginary and real components of the plot, respectively. The small semi-circle in the high-frequency region corresponds to a charge transfer resistance (R_ct_) of 0.48 Ω, which signifies efficient electron transfer at the electrode-electrolyte interface. The x-intercept at the high-frequency region shows an equivalent series resistance (R_s_) of 3.70 Ω, suggesting low intrinsic resistance, minimal electrolyte resistance, and efficient contact between the VACNTs and the Cu current collector. The values of R_ct_ and R_s_ exhibited by the electrode are due to the direct attachment of the electroactive VACNTs to the Cu current collector. Notably, the R_ct_ value obtained in this study is lower than many previously reported for CNT-based EDLC electrodes fabricated using the traditional methods. Zhang et al. [64] for example, prepared a CNT electrode via a cut–paste technique on a nickel foam substrate. Although their electrode delivered a slightly higher specific capacitance, the Nyquist plot revealed a pronounced semicircle in the high-frequency region, indicative of a higher charge transfer resistance than observed in our directly grown VACNT–Cu electrode. In another study, Yan et al. [65] investigated various carbon-based slurry formulations for flow-electrode capacitive deionization, reporting R_ct_ values of 6.11 Ω (AC), 5.26 Ω (AC/CB), 4.34 Ω (AC/CNT), and 2.61 Ω for the optimized AC/(CB + CNT) composite. Despite improvements from enhanced conductive networks, their best-performing slurry still exhibited a substantially higher interfacial resistance than our electrode. Azam et al. [66] and Huq et al. [54] employed CNT-enhanced activated carbon electrodes using drop-casting and electrophoretic deposition, respectively, but their Nyquist plots displayed broader semicircles in the high-frequency region, indicating slower charge transfer. Similarly, electrodes developed by Azam et al. and Huq et al. using drop-casting and electrophoretic deposition techniques, respectively, showed broader semicircular arcs in the high-frequency domain of their impedance spectra, reflecting sluggish charge transfer kinetics. These comparisons reinforce the benefit of the direct VACNT–Cu interface employed in our study, which ensures efficient electronic pathways and reduced interfacial resistance without the need for additional conductive additives or binders. Additionally, in the low-frequency region, the plot shows a near-vertical line indicative of the Warburg resistance (W). The presence of the Warburg resistance suggests a diffusion-controlled process, typical of EDLCs, where the ions must penetrate deep into the porous structure of the electrode during charging and discharging cycles [67]. The nearly vertical nature of the Warburg line indicates favorable ion diffusion properties, as the electrode can maintain a capacitive response with minimal resistive losses due to the efficient movement of ions within the porous VACNT network, allowing for effective charge storage. The inset of Figure 5a is the equivalent circuit model of the Nyquist plot.

The capacitance retention property of the VACNT-based electrode was investigated by repeating the CV measurements at 20 mV/s for 3000 cycles. Figure 5b shows the electrode exhibiting high cycling stability with 92% capacity retention after 3000 cycles. The inset of Figure 5b reveals a slight deviation in the CV curve during the first cycle, likely due to the initial conditioning of the electrode [68]. However, subsequent cycles show near-overlapping curves, indicating stable and consistent electrochemical behavior with minimal performance variation as cycling progresses. The GCD curves at 0.2 A $g^{-1}$ before and after the cyclability test shown in Figure 5c reveal a slight decrease in discharge time after cycling, indicating a minor loss in capacitance from 2.20 F g^−1^ to 2.03 F g^−1^. Despite this, the overall shape of the curves remains largely unchanged, reflecting the electrode’s durability and suitability for long-term use.

The Ragone plot shown in Figure 5d illustrates the relationship between the energy and power densities for the VACNT-based electrode. The values were calculated using Equations (3) and (4) below [67]:

(3) $$E\left(Wh\;Kg^{-1}\right)=\frac{1}{2}\frac{C_{\mathrm{grav}}V^{2}}{3.6}$$

(4) $$P\left(W\;Kg^{-1}\right)=\frac{E\times 3600}{\Delta t}$$

where V is the potential window during the discharge process, C_grav_ is the specific capacitance from the GCD curves in Figure 4c, and Δt is the discharge time. The electrode achieves an energy density of about 0.27 Wh K$g^{-1}$ at the minimum power density. However, as the power density increases, the energy density decreases, reaching approximately 0.20 Wh K$g^{-1}$ at the maximum power density of 450 W K$g^{-1}$. This trend is typical of supercapacitors, where high power output leads to faster charge–discharge cycles, reducing the time available for full energy storage. The moderate decline in energy density at increasing power density suggests that the VACNT-based electrode maintains a good balance between energy storage and power delivery, making it suitable for applications where high power density is crucial without sacrificing significant energy storage capability.

## 4. Conclusions

Direct growth of VACNTs on Cu substrates via PECVD has proven to be an effective method for fabricating high-performance EDLCs. The VACNT-based electrode demonstrates low equivalent series resistance (3.72 Ω) and charge transfer resistance (0.48 Ω), contributing to efficient electron transfer. Additionally, the electrode exhibits excellent cycling stability, retaining 92% of its initial specific capacitance after 3000 cycles, highlighting its durability and suitability for long-term energy storage applications. Beyond electrochemical performance, the fabrication method offers significant advantages over conventional approaches. By eliminating the need for metal catalysts, surface treatments, and polymeric binders, this binder-free, catalyst-free process simplifies electrode preparation and enhances structural integrity. The direct growth of VACNTs on untreated Cu foil ensures strong nanotube-substrate contact, reducing interfacial resistance. However, the relatively low specific capacitance remains a limitation, primarily due to the intrinsic hydrophobicity of CNTs, which hinders interaction with aqueous electrolytes and limits ion accessibility. To overcome this, future studies will focus on the surface functionalization of the VACNTs and their evaluation in non-aqueous electrolytes, which are expected to improve the capacitance while preserving the low interfacial resistance demonstrated here.

## Data Availability

The original contributions presented in this study are included in the article. Further inquiries can be directed to the corresponding author.
